# Supplementary figures and images for: Transmission of H9N2 Low Pathogenicity Avian Influenza Virus (LPAIV) in a Challenge-Transmission Model
Source: Vaccines (Basel). 2022 Jun 28;10(7):1040. doi: 10.3390/vaccines10071040 (PMC9316524; doi:10.3390/vaccines10071040)

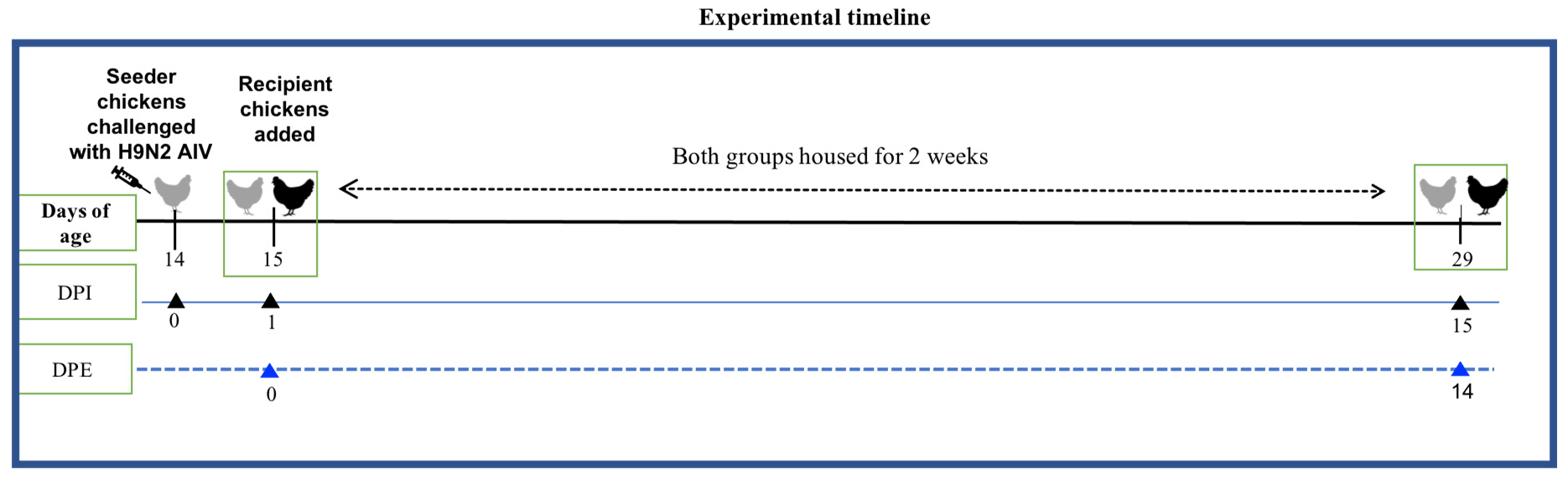

Supplement: Supplementary file 1 [file vaccines-10-01040-s001.zip › vaccines-1731764-Figure S1.png]
